# Umbilical cord care in Ethiopia and implications for behavioral change: a qualitative study

**DOI:** 10.1186/1472-698X-14-12

**Published:** 2014-04-18

**Authors:** Yared Amare

**Affiliations:** 1 Consultancy for Social Development, Addis Ababa, Ethiopia

**Keywords:** Umbilical cord care, Newborns, Infection, Ethiopia

## Abstract

**Background:**

Infections account for up to a half of neonatal deaths in low income countries. The umbilicus is a common source of infection in such settings. This qualitative study investigates practices and perspectives related to umbilical cord care in Ethiopia.

**Methods:**

In-depth interviews (IDI) were conducted in a district in each of the four most populous regions in the country: Oromia, Amhara, Tigray and Southern Nations, Nationalities and Peoples Region (SNNPR). In each district, one community was purposively selected; and in each study community, IDIs were conducted with 6 mothers, 4 grandmothers, 2 Traditional Birth Attendants and 2 Health Extension Workers (HEWs). The two main questions in the interview guide related to cord care were: How was the umbilical cord cut and tied? Was anything applied to the cord stump immediately after cutting/in the first 7 days? Why was it applied/not applied?

**Results:**

The study elucidates local cord care practices and the rational for these practices. Concepts underlying cord tying practices were how to stem blood flow and facilitate delivery of the placenta. Substances were applied on the cord to moisturize it, facilitate its separation and promote healing. Locally recognized cord problems were delayed healing, bleeding or swelling. Few respondents reported familiarity with redness of the cord - a sign of infection. Grandmothers, TBAs and HEWs were influential regarding cord care.

**Conclusions:**

This study highlights local rationale for cord practices, concerns about cord related problems and recognition of signs of infection. Behavioral change messages aimed at improving cord care including cleansing with CHX should address these local perspectives. It is suggested that HEWs and health facility staff target mothers, grandmothers, TBAs and other community women with messages and counseling.

## Background

More than 3 million neonatal deaths occur every year, amounting to 40% of deaths in children younger than 5 years of age [1]. In Ethiopia, neonatal mortality has remained stable at around 37 deaths per 1000 live births in recent years [2]. In settings where the majority of deliveries occur at home, often under sub-optimal and unhygienic conditions, infections account for half of neonatal deaths [3,4]. The umbilicus is an important source of infection in the first few days of life due to unhygienic cord care practices including cord cutting and tying [5,6]. Studies from a range of countries show that various substances including cow dung, ash, mud, rat feces, turmeric, oil and butter are commonly applied on the umbilical wound in order to promote healing [7-9]. These practices are associated with an increased risk for omphalitis which is directly associated with increased mortality [10-12].

To eliminate the incidence of omphalitis and subsequent mortality, improved cord care practices such as hand washing, the use of a new blade to cut the cord and hygienic cord tying have been widely promoted. The World Health Organization has also recommended dry cord care as well as the use of topical antiseptics in settings with a high risk of infection [5].

Programmatic interventions aiming to reduce neonatal morbidity and mortality by promoting improved cord care practices are challenged by local practices and beliefs. A better understanding of such practices and beliefs is important for designing effective behavioral change strategies. This study sought to answer the following research questions: 1) What are the local perspectives on cord care and cord related problems? 2) What are the implications of these perspectives for behavioral change strategies?

## Methods

### Study area

Data were collected from one district in each of the four most populous Federal Regions in Ethiopia - Oromia, Amhara, Tigray and Sidama Zone in Southern Nations, Nationalities and Peoples Region (SNNPR). Within each district one community was slected using criteria such as distance from urban areas and health centers, accessibility and availability of HEWs (Health Extension Workers). The residents of the districts from which study communities were selected are predominantly agriculturalists who combine cereal production and animal husbandry, except in the Sidama Zone where instead of cereal production, *enset* or the false banana plant is cultivated. The majority of community members are subsistence producers and most of the adult population is illiterate. The study communities are located between 10 to 30 kms from an urban center and between 3 to 10 kms from a health center.

### Data collection and analysis

The data on which this article is based were collected as part of formative research conducted to investigate the potential for the use of chlorohexidine for cord care in Ethiopia [Amare Y, unpublished report]. The study took place between January and May 2013 with data collection occurring in March and April, 2013. Qualitative methods, comprising of in-depth interviews (IDIs), were used in this study as they are best suited to investigate practices and beliefs in a holistic and naturalistic fashion. In-depth interviews allow us to collect detailed information on respondents’ perspectives including their behavior, beliefs, knowledge and sensory responses [13]. The interviews were based on semi-structured guides “see Additional file 1”. The guides included questions on conditions of delivery, immediate newborn care, cord care practices, sources of advice on cord care, perceptions of cord-related problems and familiarity with cord infection. To assess their recognition of cord related infection, respondents were shown a picture of a baby with redness on the cord and were asked if they had seen such a condition before. A component of the guide, not reported on in this article, included questions on attitudes to the potential introduction of Chlorohexidine (CHX) for cord cleansing.

In each of the four study communities, IDIs were conducted with 6 mothers, 4 grandmothers, 2 Traditional Birth Attendants (TBAs) and 2 Health Extension Workers (HEWs). TBAs were community women, usually untrained, who had significant experience in helping women deliver at home. HEWs were female health workers with 10 to 12 years of schooling and one year of training in community health who operate at the community level within the national Health Extension Program. They mainly provide health education but also some curative services. The respondents who were selected as participants in the study were closely involved in newborn care which enabled them to provide accurate information. Sample sizes for each respondent category were determined based on the number of interviews expected to capture the variety of respondent practices and perspectives and to reach saturation point (i.e. sampling until no new information emerged) . The numbers of TBAs and HEWs in each community was small and the sample included most or all available respondents in a community.

Study participants were selected purposively from different households with the involvement of community facilitators. To enhance recall, mothers, grandmothers and TBAs who had been involved in a delivery in the past three months were selected. To represent varying perspectives and practices within a community, fieldworkers selected mothers from different age and educational levels. The limited number of women in a community who had delivered in the last three months restricted the use of additional criteria to select participating mothers, but fieldworkers paid attention to getting a range of parities and socio-economic status where possible. Grandmothers and TBAs were predominantly older and illiterate (see Table 1 in Results section).

**Table 1:** Socio-demographic background of study participants

	**Mothers**	**Grandmothers**	**TBAs**	**HEWs**
**Age**				
< 20	3			
20-30	12		1	6
31-40	8	2	1	2
>40	1	14	6	
**Parity**				
1-2	9			
3-4	6			
5-8	8			
>8	1			
**Education**				
Illiterate	18	16	5	
Literate			2	
1-4^th^ grade	4			
5-8^th^ grade	1		1	
10^th^ grade	1			
10 - 12^th^ grade + 1 year in community health				8
**Total**	24	16	8	8

Two research teams, each consisting of two experienced qualitative interveiwers, were trained to conduct the interviews. The training workshop included role play sessions which were also used to fine-tune the interview guides. Each team conducted the interviews in two study communities. The interviews were recorded with the use of a digital voice recorder and field notes. Expanded notes were developed from the recordings and fieldnotes just after the interviews. Such notes capture as much detail as possible as well as the voice of the respondent including verbatim quotations and locally relevant language terms with direct translations. Responses are put in context through interviewer comments and observations regarding the informant and interview situation.

The reliability of data collection was enhanced through rigorous training of the interviewers on study objectives and tools and through repeated role plays. Various steps were taken to ensure the trustworthiness of the data that were collected. Respondents were assured that the interviews were being conducted purely for research purposes. The interviewers were experienced and had the ability to encourage and assess the authenticity of responses based on consistency of responses within an interview as well as the interview dynamics. Collecting data from an adequate number and from different types of respondents allowed the use of triangulation to assess the consistency and trustworthiness of the data. The largely similar responses related to reported practices within and between respondent groups and the internal consistency of most interviews lead us to believe that the data are trustworthy. Divergent responses, which usually reflect individual differences, were always noted. The data on perspectives is less prone to reporting bias.

Data analysis was content based and descriptive. The expanded notes were coded using the qualitative software program, Nvivo. The list of folders and nodes (themes) to be used for coding were developed on the basis of the questions in the interview guides and on expected responses based on previous studies. This list was supplemented with additional nodes or themes after reviewing the expanded notes and during the coding process. The data within each theme were reviewed, synthesized descriptively and written-up. The prevalence of responses and differences by region and respondent category are noted where relevant. Respondent statements which are especially expressive, informative and representative have been selected and presented as quotes.

### Ethical approval and consent

Ethical approval was obtained from the Ethiopian Health and Nutrition Research Institute. We received permission for the study from the Federal Ministry of Health and Regional Health Bureaus. Study participants were informed about the objectives of the study and assured that their participation and responses were anonymous and confidential. Each participant gave her oral consent to be interviewed.

## Results

### Study participants

Table 1 shows some socio-demographic characteristics of the study participants. The majority of mothers were 20 to 40 years of age, whereas grandmothers and TBAs are mostly older than 40. Most of the HEWs were 20 to 30 years old. A high proportion of mothers have 5 or more children. The majority of participants from the community are illiterate, whereas the HEWs have had at least 11 years of schooling.

### Umbilical cord tying and cutting

Mothers were asked about the care provided to the umbilical cord after their most recent delivery, almost all of which took place at home with a few women who were taken to a health facility after delivery. Of the 24 mothers interviewed, 22 reported that the umbilical cord was cut with a new razor blade which was sometimes boiled before use. A mother from the Oromo community used an old blade and a mother from the Sidama community used a knife which was normally used for cutting *kocho -* a foodstuff produced from the false banana plant. There was more variability in what was used to tie the cord with sewing thread, the thread from kerosene stoves, sisal thread, or thread or strips of cloth from a local blanket, traditional shawl or bed sheets all used. HEWs typically used sterilized thread and scissors to tie and cut the cord.

The cord was usually cut immediately after birth and before the delivery of the placenta, but in Sidama, it was sometimes cut after its delivery. All HEWs, except one in Oromia, also reported that they cut the cord before the delivery of the placenta. Cord cutting and tying practices varied by study communities (see Table 2). The cord was cut at a length of one or two joints of a finger, or up to a whole finger in all communities except the Amhara community. It is usually tied only on the baby’s side in the Oromo and Sidama communities, on the side of the placenta in the Amhara community, on both sides in Tigrai community, or not tied at all in Sidama. HEWs generally tied the cord twice - two finger widths from the baby and again another two finger widths away – and then cut it in the middle. The exception was the HEW in Sidama who tied it only on the side of the baby. In all of the study communities, there was variability in whether the cord was bathed during routine bathing.

**Table 2:** Community practices and perspectives related to cord care

**Practices and perspectives**	**Oromia**	**Amhara**	**Tigray**	**Sidama**
**Length of cord cut**	1 or 2 finger joints, 1 whole finger	1 or 2 finger joints	1 or 2 finger joints, 1 whole finger	1 or 2 finger joints, 1 whole finger
**Cord cutting in relation to delivery of placenta**	Before delivery of placenta	Before delivery of placenta	Before delivery of placenta	Before or after delivery of the placenta
**Cord tying**	On the side of baby	On the side of the placenta	On both sides of cut	On the side of baby or not tied
**Reasons for cord tying**	Stem blood flow, Ensure survival of baby and mother, Prevent air from entering baby	Stem blood flow, Facilitate delivery of placenta	Stem blood flow, Ensure survival of baby and mother, Prevent placenta from returning, Culture	Stem blood flow
**Substances applied on cord**	Butter, Petroleum jelly, Hair lotion	Butter, Nothing	Butter	Petroleum jelly, Hair lotion, Nothing
**Timing of application**	After cutting cord	2^nd^ or 3^rd^ day	After cutting cord	2^nd^ or 3^rd^ day
**Reasons for application**	Soften the cord, Promote cord separation	Soften the cord, Prevent sores, Promote healing, Promote cord separation	Soften the cord, Prevent sores, Promote healing	Prevent sores, Promote healing, Promote cord separation
**Reasons for delaying or avoiding application**	Advice from HW, Cord is moist on 1^st^ & 2^nd^ day	Unnecessary, Custom, Advice from HW, Cord is moist on 1^st^ & 2^nd^ day	N/A	Custom

Different reasons were given for tying the cord, which differed somewhat by region: to facilitate delivery of the placenta in Amhara, to stem blood flow in all regions, to ensure survival of the baby or mother in Oromia and Tigray, to prevent air from entering the baby in Oromia, to prevent the placenta from going back inside or simply because it is culturally prescribed in Tigray (Table 2). Thus, a 65 year old grandmother in the Amhara region explained that the cord is tied, “so that the blood pulls the placenta out. The one that is on the baby’s side is not tied since it is sealed already.” On the other hand, a 38 year old mother in Tigray, where the cord is tied on both sides of the cut, said, “The cord will be tied before it is cut because blood will flow from the placenta to the baby and may create a problem, and blood will also flow out from her.” A 25 year old mother in Oromia stated “The baby will die if the cord is not tied properly.” HEWs also generally believed that leaving the cord untied may lead to bleeding and even death of the baby.

### Application of substances

Nineteen of the 24 mothers applied something to the cord just after cutting it or in the first few days after birth. Butter was universally applied in Amhara and Tigray and commonly applied in Oromia, whereas some mothers in Sidama and Oromia reported that petroleum jelly and hair lotion were applied. Such substances were usually applied just after the cord was cut in Oromia and Tigray, whereas application mostly occured on the second or third day in Amhara and Sidama (Table 2). The two mothers who reported that nothing was applied to the cord were from the Amhara and Sidama communities. HEWs mostly do not apply anything to the cord except for HEWs in Oromia and Tigray who said that they apply iodine and Gensen Violet, respectively.

Grandmothers, mothers of newborns, TBAs or neighbors were the ones who applied substances on the cord. Substances were usally applied gently with the tip of a finger and handwashing before application was rare and was not a community norm. The most frequent practice was to apply the substance to the base of the cord and the area surrounding it, although some mothers in all four study communities also applied it on the protruding part and tip of the cord. The application of substances usually continued until the cord separated or up to 7 or more days after birth. Application was conducted one to two times a day in Amhara and Oromia, three times a day in Sidama and one to three times a day in Tigray, usually after bathing the base of the cord.

A number of closely related reasons were given for applying substances on the cord (Table 2). The most frequently mentioned reason, especially in Oromia, Amhara and Tigray, was to soften or moisturize the cord, especially at its base where it is expected to break off. A 22 year old mother in Oromia said “It protects the cord from drying and hurting. It makes the cord soft.” A 38 year old mother in Tigray also stated “If we do not apply butter on the cord and it dries up, it will be painful to the baby.” Another 22 year old mother in Oromia said “We apply butter on the cord so that it does not get dry and hurt the baby by sticking to his clothes.” The HEWs in Oromia and Tigray who applied iodine or GV to the cord on the first day after birth explained that they did so to prevent bleeding or infection. The latter said “We pour GV from the package on to the tip of the cord without touching it with our hands so that the cord dries and blood does not flow out.”

The substances applied were also believed to moisturize the cord to prevent sores and promote healing. This reason was mentioned mostly by mothers and grandmothers in Amhara, Tigray and Sidama. A 55 year old grandmother in Sidama said, “We apply vaseline to the base of the baby’s cord so that the skin around it does not shrivel and heals sooner.” The desire to loosen up and promote healing of the skin at the point where the cord will drop off was a recurring reason for applying substances to the base of the cord. A 65 year old grandmother in Amhara elaborated: “We apply butter on the bottom of the cord so that it will be soft and not tear up. Instead, new skin will grow in the space that opens up as the cord drops off.” A closely related and commonly mentioned reason for moisturizing the base of the cord was to speed up cord separation. A 45 year old mother in Amhara explained “When I apply butter on the edge of the cord, it will fall off by itself when it is moisturized from the inside.”

The perception that the cord is moist in the first one or two days after birth was why some respondents in Amhara and Oromia delayed applying substances on it (Table 2). A 55 year old grandmother in Oromia stated “Nothing is applied on the first day since the cord is still moist. The application starts from the second day onwards.” A 30 year old mother in Amhara who started applying butter only on the third day also said, “The baby is wet the day he is born. It is when the cord dries that we apply butter so that it falls off.” Those who did not apply anything to the cord at all in Oromia, Amhara and Sidama explained that it is not customary, that it is unnecessary or that they have been advised against it by health workers.

### Sources of advice on cord care

Various sources of information on cord care were revealed by the study. Many mothers indicated that they learnt about cord care through observation or their exposure to local customs. A 25 year old mother in Amhara, stated “No one gave me advice. I applied what I observed when my mother applied butter and washed the cord.”

Another important source of information on cord care was the advice provided by grandmothers, neighbors, TBAs and HEWs. Grandmothers in all sites except Sidama commonly advised mothers to apply butter on the cord. A 22 year old mother in Oromia stated, “My mother told me to apply butter to the cord stump after each bath in order to prevent it from sticking to clothes.” Grandmothers, neighbors, TBAs and fathers also advised mothers on hygiene including the need to bath newborns’ cords, bodies or clothes regularly. A 56 year old grandmother in Amhara said “I advise her not to sleep in an unclean place, to change her baby’s clothes every day, and to bath the baby.” A 60 year old TBA in Sidama said, “I tell her that the cord stump drops off on the seventh day so she needs to keep washing the base of the cord with soap and water.” Most HEWs advised mothers not to apply anything on the cord and to keep it dry in order to prevent infection, except one in Sidama who advised them to apply tetracycline on the cord. An HEW in Amhara said, “We tell them that they should not apply anything on the cord and that it will fall off even if it becomes dry.” Only two HEWs reported advising mothers to bath the cord, to avoid pulling the cord during delivery or to go to a health facility in case of an infected cord.

### Cord related risks and infections

Local understandings of risks and infections affecting the cord inform people’s efforts to prevent and respond to them. Many mothers, grandmothers and TBAs were not aware of any risks or problems affecting the cord. Those who were aware described sores, a failure to heal properly and bleeding. Such problems are believed to arise when the cord becomes dry or sticks to clothes, especially if butter is not applied, or when the cord is not bathed. A 30 year old mother in Amhara said, “The cord may develop sores. If we do not bath and massage the cord with butter until it is removed, the base of the cord may crack and bleed.” The other condition that was recognized by a mother and grandmother in Amhara and Oromia, respectively, is swelling of the cord which they believed occured when the cord is not cut properly or if it is pulled. The grandmother in Oromia explained “If they don’t cut the umbilical cord with proper care and pull it while tying it, the cord can swell.”

Only one mother and one TBA in Oromia, one grandmother in Tigray and one HEW in Sidama acknowledged seeing redness of the cord as depicted in the picture they were shown, whereas the vast majority of respondents had not seen it before. The response of a 38 year old grandmother in Amhara was typical of the latter group: *“*I have never seen such redness on my baby or on others”. The three women who were aware of it believed that it is caused by lack of hygiene or pulling of the cord. The mother explained “When the baby is not bathed daily and his cord is not kept clean, it will turn out to be reddish like this”. The grandmother and TBA agreed that it occurred when the cord is pulled. The former said “It happened because the cord was not cut and tied by a capable woman. She pulled the cord when she cut and tied it.” The HEW who had encountered the condition believed that it was caused due to the failure to cut the cord at a correct length or the use of a rusty blade to cut the cord. Although they had not seen redness of the cord previously, the other HEWs recognized that it was a sign of infection which was caused by lack of hygiene and application of substances or a condition which arose when the cord was pulled or not tied or cut properly. The women who recognized the condition as well as most of the HEWs said that they would take or refer a baby who has it to a health center or doctor.

## Discussion

This study has described practices and perspectives related to cord care in communities selected from four of the major regions in Ethiopia. The findings of this study contrast with those from other similar studies. The predominant use of a new razor blade and cutting of the cord before the emergence of the placenta in Ethiopia is in contrast to the relatively common use of old razor blades and cutting of the cord after the emergence of the placenta reported in Pakistan and Bangladesh respectively [6,7]. The length and location at which the cord was cut was variable across the study communities and was also variable in other countries. The practice of not tying the cord at all or only on the side of the baby is only common in certain regions of Ethiopia [7]. The prevalence of the practice of applying substances on the cord in Ethiopia ranks is high compared to levels in Nepal, Bangladesh and Pakistan [7,10,12]. The use of butter, petroleum jelly and hair lotion for this purpose in Ethiopia contrasts with the use of a wide variety of substances such as cow dung, mustard oil, turmeric, boric powder, ash and mud in other countries. The local rationales for cord practices are generally not reported in other studies and this study has attempted to fill this gap.

Behavior change strategies need to address the local rationales for cord care practices in order to effectively bring about improved practices such as optimal cord tying and cutting, avoiding application of substances on the cord and hand washing [5,14,15]. For instance, in addition to explaining the rationale for tying the cord on both sides of the cut, behavior change messages should address local conceptions regarding the role of cord tying in stemming blood flow and facilitating delivery of the placenta. Similarly, messages discouraging application of substances on the cord should address local perceptions regarding the role of such substances in moisturizing the cord and promoting its healing and separation. We also need to consider local concerns about cord related problems such as sores, bleeding and cracking when designing messages aimed at promoting dry cord care and better hygiene.

Pre-existing community awareness of cord related infection enhances receptivity to improved cord care practices directed at reducing such infection. Study findings show that few respondents had previously encountered redness of the cord. Several reasons may account for this: relatively low rates of infection in the study communities and infections not showing as redness. The sign of infection shown to respondents was restricted to redness of the cord and did not include pus, or swelling which may have been recognized by more respondents. It is therefore important to expand awareness of infection and its various signs in order to strengthen behavioral change for improved cord care. It would also be very useful to conduct studies on those families who have actually experienced infection of the cord to gain insight into their cord care practices, their perceptions of the causes of infection and responses to it.

Messages on improved cord care would be most accessible to rural communities in Ethiopia if they are provided by HEWs during their home visits for antenatal and post-natal care and by health facility staff who provide delivery services. Since mothers, grandmothers, TBAs, other community women and HEWs participate in or provide advice for cord care, it is important to target them with behavioral change messages and to strengthen the counseling skills of HEWs on cord care.

Studies conducted in Nepal, Bangladesh and Pakistan have shown that cleansing the cord with chlorohexidine (CHX), a widely used antiseptic, significantly reduces incidence of omphalitis and mortality in newborns by [16-18]. This investigation of local cord care practices was part of a formative study on the potential for the use of CHX for cord care in Ethiopia. Another component of the study looked at aspects related to the potential introduction of CHX in Ethiopia including receptivity to it and preferences regarding modes of introducing it. All respondents except one expressed willingness to use a product that would prevent infection of the cord, which indicates that there is strong potential for promoting CHX for cord care in Ethiopia. The effectiveness of messages which promote CHX for cord cleansing can be stronger if they incorporate perceived benefits of substances traditionally applied on the cord such as moisturizing and softening the cord and promoting its healing, in addition to its benefits in preventing infection. Since cleansing with CHX has been found to prolong cord separation slightly, messages and counseling on CHX should also emphasize that this is not harmful to the newborn and indicates that it is keeping the cord clean [19,20].

The findings of this study are based on data from a community in each of four of the major regions in Ethiopia. Although they are not representative of these or other regions, the findings are indicative of the diversity of cord related practices and perspectives in the country. Therefore, they can provide the basis for designing behavioral change strategies which can be adapted to local and regional variations in cord care through further research.

## Conclusion

This study has investigated local perspectives regarding cord care in several Ethiopian communities to shed light on rationales for cord care practices, concerns about cord related problems and familiarity with cord redness as a sign of infection. To be more effective in improving cord care practices, behavioral change messages should address such rationales, concerns and awareness of infection. HEWs and health facility staff should target mothers, grandmothers, TBAs and other community women with such messages and counseling during home visits and facility deliveries. Messages promoting CHX for cord cleansing should incorporate locally desired outcomes with respect to the cord in addition to its prophylactic benefits.

### Limitations of the study

The data for this qualitative study were collected from single communities selected purposively from each of the four regions. The findings are therefore indicative and not representative of the situation in the regions concerned. The data are based only on the reported statements of respondents. Although there is no reason to doubt their validity, the statements have not been further verified through observation.

## Abbreviations

CHX: Chlorohexidine; HEW: Health extension worker; IDI: In-depth interview; SNNPR: Southern Nations, Nationalities and Peoples Region; TBA: Traditional birth attendant.

## Competing interests

The author declares that he has no competing interests.

## Authors’ contribution

YA designed the study, trained and supervised data collectors, analyzed the data and wrote the manuscript.

## Authors’ information

YA has a Ph.D. in anthropology. He is a research consultant and manager of the Consultancy for Social Development. He specializes in qualitative and social research on such issues as maternal and child health, reproductive health and nutrition.

## Pre-publication history

The pre-publication history for this paper can be accessed here:

http://www.biomedcentral.com/1472-698X/14/12/prepub

## Supplementary Material

Additional file 1 In-depth interview guides. Click here for file
