# Supplementary material for: Umbilical cord care in Ethiopia and implications for behavioral change: a qualitative study
Source: BMC Int Health Hum Rights. 2014 Apr 18;14:12. doi: 10.1186/1472-698X-14-12 (PMC4021177; doi:10.1186/1472-698X-14-12)
Supplement: Additional file 1 — In-depth interview guides. [file 1472-698X-14-12-S1.docx]

**In-depth Interview Guide: Mothers**

# 1: Socio-demographic and interview information

| - 1. ID:   2. Interview date:   3. Interview start time:   4. Interview end time:   5. Place of residence:   6. Marital status:   7. Region:   8. Religion:   9. Education:   10. Main occupation: | - 1. Mother’s age:   2. Total number of children delivered:   3. Main birth attendant:   4. Place of delivery:   5. Sex of baby:   6. Date of birth:   7. Socio-economic status (observe):   8. Interviewer code:   9. Tape recording number: |
| --- | --- |

**2: General care of the newborn**

2.1 What are the things that you have done to keep your baby healthy in the first month of life?

**3: Delivery and umbilical cord care**

3.1 Please tell me what happened during your most recent delivery?

Who was present and what role did each person play?

3.2 Can you describe what was done with the baby immediately after delivery?

- 1. How was the umbilical cord cut and tied?
  2. Was anything applied to the cord stump immediately after cutting? What was applied? Who applied it? How was it applied? Why was it (not) applied?
  3. What was applied to the cord stump over the next 7 days? When did application start? How frequently? Who applied it? How was it applied? Why was it (not) applied? Was anything applied on the rest of the body? **Inquire about its relation to application on the cord**
  4. Where do you get the substance applied to the cord stump during the first seven days?
  5. What characteristics of the substance applied on the cord do you like? What characteristics do you dislike?
  6. How else was the cord stump cared for in the first 7 days?

**Probe:**

**-** Cleanliness, Cover

3.9 Who gave advice on caring for the cord stump? What advice did they give? **Probe for** family members, health workers or other community members

3.10 What are the risks related to the cord in the first 7 days?

3.11 **SHOW A PICTURE OF THE CORD**:

- - 1. Have you ever seen redness around the umbilical cord like in the illustration?
    2. What causes this condition?
    3. Is it a problem? Why?
    4. How can you prevent this condition?
    5. What will you do if your baby has this condition?

**4. Interviewer comments**

**In-depth Interview Guide: Grandmothers**

# 1: Socio-demographic and interview information

| - 1. ID:   2. Interview date:   3. Interview start time:   4. Interview end time:   5. Place of residence:   6. Marital status:   7. Region:   8. Religion:   9. Education:   10. Main occupation: | - 1. Age:   2. Socio-economic status (observe):   3. Interviewer code:   4. Tape recording number: |
| --- | --- |

**2: General care of the newborn**

2.1 What are the things that you have done to keep a baby healthy in the first month of life?

**3: Delivery and umbilical cord care**

3.1 Please tell me what happened during the most recent delivery you attended?

Who was present and what role did each person play?

3.2 Can you describe what was done with the baby immediately after delivery?

- 1. How was the umbilical cord cut and tied?
  2. Was anything applied to the cord stump immediately after cutting? What was applied? Who applied it? How was it applied? Why was it (not) applied?
  3. What was applied to the cord stump over the next 7 days? When did application start? How frequently? Who applied it? How was it applied? Why was it (not) applied? Was anything applied on the rest of the body? **Inquire about its relation to application on the cord.**
  4. Where do you get the substance applied to the cord stump during the first seven days?
  5. What characteristics of the substance applied on the cord do you like? What characteristics do you dislike?
  6. How else was the cord stump cared for in the first 7 days? Who gave such care?

**Probe:**

**-** Cleanliness, Cover

3.9 Who gave advice on caring for the cord stump? What advice did they give? **Probe for** family members, health workers or other community members

3.10 What are the risks related to the cord in the first 7 days?

3.11 **SHOW A PICTURE OF THE CORD**:

- - 1. Have you ever seen redness around the umbilical cord like in the illustration?
    2. What causes this condition?
    3. Is it a problem? Why?
    4. How can you prevent this condition?
    5. What will you do if a baby has this condition?

**4. Interviewer comments**

**In-depth Interview Guide: Traditional Birth Attendants**

# 1: Socio-demographic and interview information

| - 1. ID:   2. Interview date:   3. Interview start time:   4. Interview end time:   5. Place of residence:   6. Marital status:   7. Region:   8. Religion:   9. Education:   10. Main occupation: | - 1. Age:   2. Socio-economic status (observe):   3. Interviewer code:   4. Tape recording number: |
| --- | --- |

**2: General care of the newborn**

2.1 What are the things that should be done to keep a baby healthy in the first month of life?

**3: Delivery and umbilical cord care**

- 1. Can you describe what you do with a baby immediately after delivery?

3.2 How do you cut and tie the umbilical cord?

- 1. Is anything applied to the cord stump immediately after cutting? What is applied? Who applies it? How is it applied? Why is it (not) applied?
  2. Do you have any involvement in the care of babies in the first 7 days after birth? How?

3.5 Do you provide any advice or assistance on the care of the cord stump? What advice or assistance do you provide?

- 1. What is applied to the cord stump over the first 7 days after birth in this community? When does application start? How frequently? How is it applied? Why is it (not) applied?
  2. What characteristics of the substance applied on the cord do you like? What characteristics do you dislike?

3.8 What are the risks related to the cord in the first 7 days?

3.9 **SHOW A PICTURE OF THE CORD**:

- - 1. Have you ever seen redness around the umbilical cord like in the illustration?
    2. What causes this condition?
    3. Is it a problem? Why?
    4. How can this condition be prevented?
    5. What is done if a baby has this condition?

**4. Interviewer comments**

**In-depth Interview Guide: Health Extension Workers**

# 1: Socio-demographic and interview information

| - 1. ID:   2. Interview date:   3. Interview start time:   4. Interview end time:   5. Place of residence:   6. Marital status:   7. Region:   8. Religion:   9. Education: | - 1. Age:   2. Interviewer code:   1.12 Tape recording number: |
| --- | --- |
|  |  |

**2: Delivery and umbilical cord care**

- 1. Have you received any training in conducting deliveries? Do you conduct deliveries? In a month, how many deliveries do you conduct?
  2. Can you describe what you do with a baby immediately after delivery?
  3. How do you cut and tie the umbilical cord?
  4. Do you apply anything to the cord stump immediately after cutting? What is applied? How is it applied? Why is it (not) applied?
  5. In the case of home deliveries, do birth attendants apply anything to the cord stump immediately before cutting? After cutting? What is applied? How is it applied? Why is it (not) applied? Was anything applied on the rest of the body? **Inquire about its relation to application on the cord.**
  6. Do you know of substances that are applied to the cord stump over the first 7 days after birth in this community? What is applied? When does application start? How frequently? How is it applied? Why is it (not) applied?
  7. Do you educate mothers or their care givers on umbilical cord care? What information do you give? When do you give them such information?

2.8 **SHOW A PICTURE OF THE CORD**:

- - 1. Have you ever seen redness around the umbilical cord like in the illustration?
    2. What causes this condition?
    3. Is it a problem? Why?
    4. How can this condition be prevented?
    5. What do you do if a baby has this condition?

**3. Interviewer comments**
